# A. V. M. A.

**Published:** 1901-08

**Authors:** 


### CORRESPONDENCE.

### A. V. M. A.

KANSAS CITY, MO., July 20, 1901.

EDITOR JOURNAL OF COMPARATIVE MEDICINE AND VETERINARY ARCHIVES:

THE plans for the Atlantic City meeting of the American Veterinary Medical Association are about completed, and the members of the Association will feel gratified to learn that such an excellent programme has been prepared for them. The local committee of arrangements have planned to make the sojourn of all who attend this meeting most enjoyable, and are determined that all shall return to their respective homes singing the praises of Atlantic City. To those who have been there it is all sufficient to name the place. Atlantic City to those who have only heard of its boardwalk, its extensive promenade, its wonderful beach and attractive ocean view, the many diversities for the tired mind and weary body, will be made to realize what it means when Atlantic City is spoken of as a pleasure resort after they attend this meeting.

The New Jersey veterinarians are exceedingly anxious that every veterinarian in America shall come to this meeting and enjoy their hospitality; they are proud of the fact that Atlantic City is located in the State of New Jersey ; they are further proud of the fact that the American Veterinary Association will be their guests at this famous resort, and they extend a hearty invitation to the veterinarians in all America to be their guests on this occasion. Not only is this invitation extended to the veterinarians, but is intended to include wives, daughters, and lady friends, special arrangements being planned for their particular entertainment.

The veterinary societies of New York and Pennsylvania have taken a special interest in this meeting, and have joined with their New Jersey confrères in an earnest effort to make the Atlantic City meeting the grandest in the history of our Association. As a special inducement to the veterinarians interested in general practice, an extensive surgical clinic has been planned, only a part of which is ready for announcement. This clinic will be held following the close of the regular meeting, the local committee having arranged for a large tent and the construction of an amphitheatre, so that all who come may have the privilege of seeing the operations in detail and to hear the explanations made by the clinicians. Some criticism has been made concerning the former clinics held in connection with the meeting of the A. V. M. A., owing to the failure of the surgeons to attend and carry out the programme. It is understood that the clinicians whose names appear below have given every assurance that they will certainly attend, and it now seems probable that none who is particularly attracted to this meeting by reason of the clinic will have occasion to express dissatisfaction. The following noted surgeons are scheduled to have a part in the clinic :

Dr. John W. Adams, Philadelphia, Pa., "Major Operations." Dr. S. J. J. Harger, Philadelphia, Pa., "Neurectomies." Dr. William J. Coates, New York, "Ligation of the Carotid." Dr. William B. E. Miller, Camden, N. J., "Ridgling Castration." Dr. Thomas G. Sherwood, New York, "Canine Ovariotomy." Dr. R. W. McCully, "Caudal Myotomy." Dr. George H. Berns, Brooklyn, "Tenectomy of the Flexor Pedis." Dr. C. E. Clayton, New York, "Median Neurectomy." Dr. A. E. Parry, New York, operation to be selected. Dr. C. C. Lyford, Minneapolis, "Operation for Bursal Enlargements."

The railroad companies operating east of Chicago and St. Louis have granted a convention rate of one and one-third fare for our meeting, which insures a low transportation, one which is still lower than the advertised excursion rates offered by the railroads leading into Atlantic City. Members who live west of Buffalo will find it advantageous to purchase their tickets to that city. For a ten-day limit the fare is a trifle more than the fare one way. Tickets with certificates can be secured from Buffalo to Atlantic City; the return to Buffalo will be at one-third of the regular rate to those who hold certificates. It is possible that at the time of our meeting a still better excursion rate will be in force from Buffalo to Atlantic City. An inquiry should be made concerning same.

In addition to the list of papers published in the July number of the JOURNAL, I can announce the following: "Some Obstructions in the Way of Efficient Meat-inspection and Milk-inspection," Dr. C. A. Cary, Auburn, Ala. "Radical Operations for Bursal Enlargements," Dr. C. C. Lyford, Minneapolis, Minn. "The Texas Fever Problem in the South," Dr. J. C. Robert, Agricultural College, Miss. "The Diagnosis of Glanders by the Strauss Method," Dr. Langdon Frothingham, Boston, Mass. "The Veterinarian on the State Boards of Health," Dr. S. B. Nelson, Pullman, Wash. "The Pathological Anatomy and Microscopical Diagnosis of Rabies," Dr. Adolf Eickhorn, Milwaukee, Wis. "Stable Hygiene," Dr. James B. Paige, Amherst, Mass.

The last-named paper will be illustrated by some sixty stereopticon views, and should prove of the greatest value to all. During the course of the meeting Dr. A. W. Bitting and Dr. R. A. Craig, of Purdue University, Lafayette, Ind., will exhibit a series of 100 photographs on comparative histology.

It is believed that our meeting in Atlantic City offers many things of great value to every veterinarian, and he who attends will be many times repaid for the expense and trouble. True it is, that clients will feel aggrieved if the veterinarian is not at hand when he calls, but he will certainly be pleased when he learns of the veterinarian's return, refreshed in mind and body, and that through association with his brother practitioners from all parts of the continent he is better able to serve the client's interests.

Yours truly,

S. STEWART,
Secretary.

---

NEW YORK STATE VETERINARY COLLEGE, CORNELL UNIVERSITY,
ITHACA, NEW YORK, July 25, 1901.

EDITOR JOURNAL COMPARATIVE MEDICINE AND VETERINARY ARCHIVES:

DEAR SIR: Your correspondent, J. P. Foster, acknowledges the justice of all I have contended for in his statement (page

455), that "it would certainly be strange that if two students of the same mental calibre, devoting an equal amount of application to their studies, were to compete for honors, that the student representing twenty-one months (twelve at 'Ontario' and nine at 'Cornell') professional study could excel the student who had spent twenty-seven months in professional study." He concedes the value of the more thorough training, and this is all that concerns us or the profession.

He is, however, conspicuously unfortunate in his choice of examples. He instanced the cases of Drs. Ryder, Howe, and Stanclift, who took prizes at "Cornell," as showing the superiority over (or equally with) "Cornell" of the "Ontario" training, and ignored altogether the fact that these men did not come into competition with anyone who had passed three years at Cornell. He also ignored the further fact that Ryder and Howe was each a graduate of a high or normal school, and thus stood for the "Cornell" principle as against the "Ontario" one.

Again, he adduces the acceptance of Dr. Fisher (the "Ontario" gold medallist), during his one and a half year of study at "Cornell" as assistant in physiology, but covers up the fact that Dr. Fisher had received an excellent education in the University of Vermont, and thus stands as a shining example of the system followed at "Cornell" as opposed to that pursued at the "Ontario Veterinary College." In quoting Fisher he condemns his whole system, as he did in quoting the cases of Howe, Ryder, and Corrigan.

As J. P. Foster apparently regrets the lack of statistics of the teachers and workers in agricultural colleges and experiment stations, I submit the following, giving the professional and literary titles of the incumbents of these positions:

| Titles. | No. |
|---|---|
| Ph.D., M.D., D.V.S. | 1 |
| M.S., with D.V.S., D.V.M. or V.S. | 4 |
| B.S., with D.V.M. or V.S. | 7 |
| M.D., with M.D.C. or V.S. | 4 |
| M.R.C.V.S., F.R.C.V.S. | 3 |
| D.V.S. | 3 |
| D.V.M. | 16 |
| V.S. | 2 |
| Vet. (Non-graduate?) | 1 |
| | 41 |

Leaving out the non-graduate we have here a showing of 2 with (apparently) V.S. alone, as compared with 38 whose titles indicate that they had the privilege of a fuller education. Precisely what was the education of these two (V.S.) I am not informed (whether they had a better preliminary education than "Ontario" requires, or had studied in Europe or elsewhere since graduation), but assuming their education to have been the barest demanded for the V.S. only, the evidence is overwhelmingly in favor of some additional requirements. There are others on the list holding the degree of V.S., but this is supplemented by other titles that show a fuller education, and thus place them in the list that speaks for higher requirements. The possession of a degree of V.S. does not make a man an example of the all-sufficiency of the lowest conditions of graduation. We must see what else he has done, and whether his success does not really witness in favor of the higher education. The bald statement that so many graduates of a given college hold important positions does not prove that the lowest requirements in that college constitute the best, or a proper, educational standard. A closer inquiry is liable to show, as in the case of the colleges and experiment stations, that the marked men are nearly all arguments for the advisability of raising the standard. J. P. Foster has virtually acknowledged this, and from what I know of the teachers in the Ontario Veterinary College I venture to say that they would prefer to have all their students enter with a preliminary education much above the lowest requirements.

Respectfully,

JAMES LAW.

---

EFFECTS OF MAMMARY INJECTIONS OF IODIDE OF POTASH. Coremans has witnessed inflammatory complication from iodide of potassium injected into the udder in the treatment of parturient paresis. These accidents, attributed to infection with the instruments or the introduction of air, may be due to an impurity or alteration in iodide of potash.

The commercial iodide is not pure, and possesses irritating properties. Beside, the solution, chemically pure, if allowed to stand, will assume a yellowish color from the formation of oxygen compounds of the iodide. Thus altered it produces irritation when injected. The solution should, therefore, be pure and freshly prepared.—*Annales de Méd. Vét.*

